# Psychometric properties of the German version of the clinician-administered PTSD scale for DSM-5 (CAPS-5) in routine clinical settings: a multi-trait/multi-method study in civilians and military personnel

**DOI:** 10.1186/s12888-025-07342-w

**Published:** 2025-10-07

**Authors:** Jan Christopher Cwik, Jan Spies, Henrik Kessler, Stephan Herpertz, Marcella L. Woud, Simon E. Blackwell, Gerd Willmund, Peter Zimmermann, Eileen Koch, Vincent Bohnacker, Benjamin Klaer, Ulrich Schnyder, Heinrich Rau, Kai Köhler

**Affiliations:** 1 Professorship for Applied Psychology, Faculty of Applied Social Sciences, Hochschule Niederrhein, Reinarzstraße 49, 47805 Krefeld, Germany; 2 Bundeswehr Center for Military Mental Health, Bundeswehr Hospital Berlin, Berlin, Germany; 3 SRH Distance Learning University, Riedlingen, Germany; 4 Department of Psychosomatic Medicine and Psychotherapy, LWL University Hospital, Ruhr-Universität Bochum, Bochum, Germany; 5 Department of Psychosomatic Medicine and Psychotherapy, Fulda Hospital, University Medicine Marburg, Campus Fulda, Fulda, Germany; 6 Department of Clinical Psychology and Experimental Psychopathology, Institute of Psychology, University of Göttingen, Göttingen, Germany; 7 Faculty of Humanities and Social Sciences, Helmut-Schmidt-Universität / Universität der Bundeswehr Hamburg, Hamburg, Germany; 8 Psychologische Hochschule Berlin, Berlin, Germany; 9 Department of Psychiatry and Psychotherapy, University of Zurich, Zürich, Switzerland

**Keywords:** Clinician-administered PTSD scale (CAPS-5), Post-traumatic stress disorder (PTSD), Psychometric properties, Diagnostic interview, Cut-off

## Abstract

**Background:**

The Clinician-Administered PTSD Scale (CAPS-5) is a structured diagnostic interview developed for diagnosing post-traumatic stress disorder (PTSD). To ensure compliance with PTSD inclusion criteria, an initial study investigated the psychometric properties and factorial structure of the German version of CAPS-5 using data collected previously. The present study’s objective was to validate the robustness of the psychometric properties of the German CAPS-5 by assessing its validity within a routine clinical context.

**Methods:**

A total of 288 participants were recruited for the study. The validity of the German CAPS-5 was assessed using a multi-trait/multi-method design. Additionally, the study explored internal consistency, test-retest reliability, interrater reliability, and the interview’s diagnostic accuracy. Ultimately, a cut-off score was determined through receiver operating characteristics curve (ROC) analyses.

**Results:**

The study demonstrated good to excellent internal consistency, test-retest reliability, interrater reliability, construct validity, and diagnostic accuracy for the German CAPS-5. Furthermore, the study established a cut-off score of ≥ 39 for the German CAPS-5 sum score.

**Discussion:**

The German CAPS-5 emerged as a structured diagnostic interview exhibiting good to excellent psychometric properties. The findings indicated solid convergent validity of the German CAPS-5; however, further research is warranted to investigate its divergent validity.

**Trial registration:**

DRKS00015325 (https://www.drks.de; registered on January 10, 2019).

## Highlights

This study aimed to examine the psychometric properties of the German version of the Clinician-Administered PTSD Scale (CAPS-5).

The German version of the CAPS-5 showed good to excellent internal consistency, test-retest reliability, interrater reliability, construct validity, and diagnostic accuracy.

Furthermore, this study established a cut-off score of ≥ 39 for the German CAPS-5 sum score.

## Background

The Clinician-Administered PTSD Scale (CAPS) [CAPS; 1, 2–4] is a structured diagnostic interview developed to diagnose post-traumatic stress disorder (PTSD) based on the criteria of the fifth edition of the Diagnostic and Statistical Manual of Mental Disorders [DSM; American Psychiatric Association [APA], 5, 6]. The CAPS is considered the diagnostic gold standard for PTSD in clinical practice and research because of its anchoring to the DSM criteria and its practicality and comparability [7–10]. CAPS is recommended by PTSD guidelines [11].

The PTSD criteria have been substantially revised since the fifth edition of the DSM [DSM-5; APA, 6]. Accordingly, CAPS was also revised to diagnose PTSD based on the criteria of DSM-5 [CAPS-5; 12]. The psychometric properties of the English version of the CAPS-5 were investigated in the context of a US military veterans sample [13]. The study revealed high interrater reliability, test-retest reliability, and internal consistency coefficients. Furthermore, the convergent validity was investigated regarding the consistency of the PTSD diagnosis based on the CAPS-5 and the DSM-IV and the association with the PTSD Checklist for DSM-5 [PCL-5; 14]. These analyses showed good discriminant validity of CAPS-5 regarding other measures regarding psychopathology (e.g., depression, anxiety, and somatization).

To date, the CAPS-5 has been translated into several languages, and the psychometric properties of these translations have been investigated [e.g., 15, 16–18]. These translations also exhibited good to excellent psychometric properties; satisfactory factorial structure model fits were reported. The first study addressing the German version of the CAPS-5 investigated its psychometric properties and factorial structure in a treatment study context [19]. That prior work combined the data of two data sets consisting of the data of participants from either a study comparing dialectic behavioral therapy for PTSD with cognitive processing therapy (study 1) [20, 21] and a study investigating emotion recognition in the context of post-traumatic stress (study 2) [22]. Using these datasets, Müller-Engelmann et al. [19] found that the psychometric properties of the German CAPS-5 are comparable to those of the English version. Furthermore, the coefficients for internal consistency and interrater reliability were high. The convergent and discriminant validity also showed similar results.

However, the data for the study of Müller-Engelmann et al. [19] had been collected in the context of other research groups that used the CAPS to validate PTSD as an inclusion criterion for their studies. The validation of the CAPS-5 and its use in clinical care were not the subjects of these studies. As the authors stated in their study [19], at the beginning of both used samples, the DSM-5 criteria for PTSD were unavailable. Thus, they used instruments to investigate the construct validity developed to assess the DSM-IV criteria of PTSD with additional items to assess the DSM-5 criteria. Accordingly, these instruments were not validated according to the DSM-5 criteria of PTSD. Furthermore, the authors stated that the construct validity of the German CAPS-5 should be investigated by using instruments that assess other constructs away from PTSD. Also, most of the participants in the study of Müller-Engelmann et al. [19] were women who experienced sexual and/or physical abuse. Finally, the interrater reliability of the German CAPS-5 was investigated based on a secondary rating of 12 video tapes, and the test-retest reliability was not investigated.

To address these limitations, further research is needed to validate the psychometric robustness of the German CAPS-5. This study aims to assess its psychometric properties and diagnostic accuracy in routine clinical settings. Specifically, it examines interrater and test-retest reliability in a diverse sample of male and female participants exposed to various potentially traumatic events. Additionally, the study evaluates the construct validity of the German CAPS-5 across a broader range of constructs. To enhance generalizability and facilitate comparisons with studies on the English version, the study includes data from both civilian and military clinical settings.

## Methods and materials

### Participants

Participants were recruited in clinical routine settings of the inpatient and outpatient clinic of the Department of Psychosomatic Medicine and Psychotherapy of the Landschaftsverband Westfalen-Lippe Universitaetsklinikum Bochum at the Ruhr-Universität Bochum and the inpatient clinic of the Bundeswehr Berlin Center for Military Mental Health (Psychotraumazentrum Bundeswehrkrankenhaus Berlin).

To be included in this study, (1) participants had to be adults (≥18 years; all genders), (2) who have experienced at least one traumatic event according to the DSM-5-A-criterion (for more details, see [6]). Furthermore, (3) the traumatic event(s) must have occurred more than a month before the application of the CAPS-5. Finally, the only exclusion criterion was an insufficient knowledge of the German language to ensure both the feasibility of the interview and the validity of the patients’ answers.

The data from two studies were used for the current study with an overall sample size of *n* = 288 participants. In the first study’s sample, *n* = 174 (60.4%) participants were recruited for the validation study of the CAPS-5 [23]. The second study sample consisted of military participants recruited to validate the PCL-5 [24] with a sample size of *n* = 114 (39.6%). Of the overall sample, *n* = 227 (78.8%) participants were members of the German army, and *n* = 61 (21.2%) participants were civilians. Furthermore, *n* = 201 participants were male (69.6%), and *n* = 73 female (25.3%). No gender-related data was available for *n* = 15 (4.8%) participants. The mean age of all participants was *M* = 37.63 years (*SD* = 9.51; Range = 19–68 years), and all participants fulfilled the A-criterion of PTSD according to the DSM-5. The frequencies of experienced traumatic events are reported in Table 1.

**Table 1:** Frequencies (percentage reported in parenthesis) of experienced traumatic events reported in the life events checklist (multiple choices possible)

Traumatic events	Happend	Witnessed	Learned about	Part of job	Not sure	Doesn’t apply
Natural disaster	47 (16.1)	33 (11.3)	27 (9.2)	32 (11.0)	5 (1.7)	148 (50.7)
Fire or explosion	82 (28.1)	49 (16.8)	17 (5.8)	34 (11.6)	3 (1.0)	107 (36.6)
Transportation accident	121 (41.4)	47 (16.1)	17 (5.8)	17 (5.8)	4 (1.4)	86 (29.4)
Serious accident	53 (18.2)	42 (14.4)	26 (8.9)	22 (7.5)	9 (3.1)	140 (48.0)
Exposure to toxic substance	30 (10.3)	2 (0.7)	8 (2.7)	13 (4.5)	25 (8.6)	214 (73.3)
Physical assault	132 (45.2)	22 (7.5)	15 (5.1)	12 (4.1)	7 (2.4)	104 (35.6)
Assault with a weapon	127 (43.5)	21 (7.2)	10 (3.4)	22 (7.5)	8 (2.7)	104 (35.6)
Sexual assault	63 (21.6)	4 (1.4)	18 (6.2)	9 (3.1)	9 (3.1)	189 (64.7)
Other unwanted/uncomfortable sex. exp.	60 (20.5)	3 (1.0)	11 (3.8)	5 (1.7)	11 (3.8)	202 (69.2)
Combat or exposure to a war-zone	151 (51.7)	4 (1.4)	6 (2.1)	30 (10.3)	1 (0.3)	83 (34.2))
Captivity	15 (5.1)	4 (1.4)	15 (5.1)	7 (2.4)	2 (0.7)	249 (85.3)
Life-threatening illness or injury	58 (19.9)	32 (11.0)	24 (8.2)	11 (3.8)	39 (13.4)	128 (43.8)
Severe human suffering	90 (30.8)	42 (14.4)	28 (9.6)	36 (12.3)	22 (7.5)	74 (25.4)
Sudden violent death	40 (13.7)	51 (17.5)	53 (18.2)	22 (7.5)	26 (8.9)	100 (34.2)
Sudden accident death	38 (13.0)	50 (17.1)	39 (13.4)	16 (5.5)	36 (12.3)	113 (38.6)
Serious injury/harm/death caused to s.o. else	33 (11.3)	7 (2.4)	15 (5.1)	20 (6.8)	52 (17.8)	165 (56.5)
Any other very stressful event or experience	95 (32.5)	12 (4.1)	14 (4.8)	25 (8.6)	30 (10.3)	116 (39.7)

Inclusion criteria were: (1) age ≥ 18 years, (2) at least one traumatic event fulfilling DSM-5 criterion A, and (3) trauma occurrence ≥ 1 month before CAPS-5 administration. The only exclusion criterion was insufficient German language proficiency.

### Procedure

Participants took part in the first assessment. In this assessment, all participants were interviewed with the CAPS-5 and asked to fill out a questionnaire battery. All participants were also asked to take part in a second assessment. If participants took part in both assessments, the second interview was conducted by a different interviewer, so the second interview was blind to the results of the first interview. All diagnostic interviews were audiotaped for post-hoc, randomly conducted quality checks of the interviews, and for the calculation of interrater reliability.

To be included in this study, (1) participants have to be adults (≥18 years; all genders), and (2) they have experienced at least one traumatic event according to the DSM-5-A-criterion. This criterion requires experiencing an event that comprises threatened death, serious injury, or sexual violation. The traumatic situation can occur to the person herself/himself, can be witnessed by the person, can happen to a close family member or friend of the person, or can include repeated or extreme exposure to aversive details of a traumatic event (for more information, see [6]). Furthermore, (3) the traumatic event(s) must have occurred more than a month before the application of the CAPS-5. There is only one exclusion criterion, i.e., an insufficient knowledge of the German language, to ensure both the feasibility of the interview and the validity of the patients’ answers.

### Study design

This study was conducted as a psychometric validation study using a multi-trait/multi-method (MTMM) framework by Campbell and Fiske [25]. The primary aim was to evaluate the psychometric properties of the German version of the CAPS-5 in routine clinical settings, with a particular focus on internal consistency, test-retest and interrater reliability, construct validity (convergent and divergent), and diagnostic accuracy. The MTMM design allowed for a structured assessment of convergent and discriminant validity across multiple instruments and measurement methods.

### Diagnostic interview and questionnaires

#### Life events checklist (LEC)

The German version [26] of the standard self-report version of the Life Events Checklist-5 [LEC-5; 27] was used for this study. The traumatic events are specified and categorized according to the kind of exposure (experienced, witnessed, learned about, exposed to aversive details). The trauma criterion of the DSM-5 was determined based on the results of the LEC-5, and the most stressful event was identified in case of more than one experienced stressful life event. This event is then used as the index trauma for the following CAPS-5.

#### CAPS-5

The German translation [28] of the CAPS-5 [12] that asks about the previous month was used for this study. A sum score for the whole interview was calculated for analysis of the validity of the CAPS-5. There was also a sum score for each subscale. Fulfillment of the PTSD diagnosis was assessed according to the scoring rules following the DSM-5. The CAPS-5 is available in three versions: (1) last month’s version, (2) last week’s version, and (3) the worst month’s version [12]. The last month’s version is the standard version that can be used to assess a current PTSD diagnosis and PTSD symptom severity [13].

For the administration of the CAPS-5, criterion A of the PTSD diagnosis according to the DSM-5 is assessed by using the LEC-5. In the next step, for each PTSD symptom, a frequency and intensity rating is conducted. For the CAPS-5 interview, the interviewer must ensure that all symptoms are evaluated concerning the index trauma. Therefore, the interviewer uses a four-point ordinal scale for the rating of the intensity (e.g., “1 = minimal” to “4 = extreme”). For the frequency rating, either the frequency itself or the proportion of time must be used (e.g., “at least twice per month” or “some of the time (20–30%”)), which depends on the rating of the item.

In contrast to former versions of the CAPS, each item is rated with a single severity score for the CAPS-5 by combining the ratings of intensity and frequency [13]. For this severity rating, a five-point ordinal scale is used (“0 = absent” to “5 = extreme/incapacitating”). All item severity scores for the CAPS-5 total symptom severity score are summed after excluding the two dissociation severity scores. For the CAPS-5 symptom cluster severity scores, the severity score of each criterion is constructed by summing the corresponding items. Finally, the PTSD diagnostic status is defined by checking each DSM-5 criterion and dichotomizing the CAPS-5 criteria according to the scoring rules (absence: < 2; presence: ≥ 2) [13].

### Questionnaires used for the investigation of the convergent validity of the German CAPS-5

An overview of all instruments, their measured constructs, their role in the MTMM framework, and the specific hypotheses for their associations with the CAPS-5 is provided in Table 2. This table serves as a roadmap for the subsequent validity analyses and complements the detailed descriptions in the following subsections.

**Table 2:** Overview of instruments, measured constructs, and hypotheses

Instrument (Abbreviation)	Construct measured	MTMM role	Expected relationship with CAPS-5	Hypothesis
PCL-5 (PTSD Checklist for DSM-5)	PTSD symptom severity (self-report)	Monotrait–heteromethod (convergent validity)	Strong positive correlation with CAPS-5 total and corresponding subscales	H1: *r* ≥ 0.70
IES-R (Impact of Event Scale–Revised, distress and frequency versions)	PTSD-related distress and frequency	Monotrait–heteromethod (convergent validity)	Strong positive correlation with CAPS-5 total score	H2: *r* ≥ 0.60
SkPTBS (Screening for complex PTSD)	ICD-11 complex PTSD symptom severity	Related trait–heteromethod (convergent validity)	Strong positive correlation with CAPS-5 total score	H3: *r* ≥ 0.60
BDI-II (Beck Depression Inventory–II)	Depressive symptom severity	Related trait–heteromethod (convergent validity)	Moderate to strong positive correlation with CAPS-5 total and D-cluster	H4: *r* = 0.40–0.70
STAI-S / STAI-T (State-Trait Anxiety Inventory)	State and trait anxiety	Related trait–heteromethod (convergent validity)	Moderate to strong positive correlation with CAPS-5 total score	H5: *r* = 0.40–0.70
DES-20 (Dissociative Experiences Scale–20)	Dissociative symptoms	Related trait–heteromethod (convergent validity)	Moderate positive correlation with CAPS-5 total score	H6: *r* = 0.40–0.60
QTF (Questionnaire of Thoughts and Feelings)	Borderline personality disorder–related cognitions	Related trait–heteromethod (convergent validity)	Low to moderate positive correlation with CAPS-5 total and D-cluster	H7: *r* = 0.20–0.40
PSWQ (Penn State Worry Questionnaire)	Pathological worry	Divergent trait–heteromethod (discriminant validity)	Low positive correlation or none with CAPS-5 total	H8: *r* ≤ 0.30
SCL-90-R GSI (Global Severity Index)	Overall psychological distress	Related trait–heteromethod (convergent validity)	Moderate positive correlation with CAPS-5 total score	H9: *r* = 0.40–0.60
SCL-90-R subscales (SOM, OBS, INT, PHO, PAR, PSY)	Somatic, obsessive-compulsive, interpersonal sensitivity, phobic anxiety, paranoid ideation, psychoticism	Divergent trait–heteromethod (discriminant validity)	Low correlations or none with CAPS-5 total	H10: *r* ≤ 0.30
BIDR-S / BIDR-I (Balanced Inventory of Desirable Responding)	Socially desirable responding (self-deceptive enhancement, impression management)	Divergent trait–heteromethod (discriminant validity)	No significant correlation with CAPS-5 total score	H11: *r* ≈ 0

#### PCL-5

The German version [29] of the PCL-5 [14] was used as a self-rating questionnaire to assess the severity of PTSD symptoms. This score was used as an external criterion for diagnostic accuracy due to the studies that recommend using a cut-off score of ≥ 33 [24]. The internal consistency of the PCL-5 for the current sample was α = 0.95 [Cronbach’s α] and ω = 0.96 [McDonald’s ω].

#### Impact of event scale-revised (IES-R)

The German version [30] of the Impact of Event Scale-Revised (IES-R) [31, 32] was used as an additional instrument to measure PTSD symptoms. The IES-R was used in two versions to assess (1) distress caused by PTSD symptoms (IES-D) and (2) the frequency of PTSD symptoms (IES-F) within the last week [33]. For the IES-F, Maercker and Schützwohl [33] provide a formula to evaluate a suspect diagnosis of PTSD (X = ((-0.02 × intrusion) + 0.07 × avoidance) + (0.15 × hyperarousal) – 4.36)). A score > 0 indicates a suspect diagnosis of PTSD. The internal consistency of the IES-R distress version for the current sample was α = 0.93 and ω = 0.93, and α = 0.90 and ω = 0.90 for the frequency version.

#### Screening for complex PTSD (SkPTBS)

The “Screening for complex PTSD” (“Screening zur komplexen Posttraumatischen Belastungsstörung”; SkPTBS) [34] is a German self-report questionnaire for the screening of complex PTSD (cPTSD) according to the 11th edition of the International Classification of Diseases (ICD-11) [35, 36]. The SkPTBS was used to assess the convergent validity of the CAPS-5 due to relatively high rates of concordance between the diagnosis of PTSD according to the DSM-5 and the cPTSD according to the ICD-11 [37, 38]. The internal consistency of the SkPTBS was calculated for the symptom severity rating items: α = 0.90 and ω = 0.90 for the current sample.

#### Beck depression inventory-revised (BDI-II)

The German version [39] of the BDI-II [40] was used to assess the severity of depressive symptoms as a secondary outcome measure. The internal consistency of the BDI-II for the current sample was α = 0.93 and ω = 0.93.

#### State-trait anxiety inventory (STAI)

The German version [41] of the state-trait anxiety inventory (STAI) [42] was used to assess state (STAI-S) and trait (STAI-T) anxiety as another secondary outcome measure. The internal consistency of the STAI-S for the current sample was α = 0.92 and ω = 0.91. It was α = 0.93 and ω = 0.92 for the STAI-T.

#### Dissociative experiences scale: 20-item short version (DES-20)

The German version [43] of the DES-20 [44, 45] was used to measure dissociative symptoms. This self-rating measure was used as a secondary outcome measure to assess the convergent validity of the CAPS-5, especially concerning the dissociative subtype of PTSD. The internal consistency of the DES-20 for the current sample was α = 0.94 and ω = 0.91.

#### Questionnaire of thoughts and feelings (QTF)

The Questionnaire of Thoughts and Feelings (QTF) is a German self-rating questionnaire to measure cognitive concepts of the bio-social model of borderline personality disorder [46, 47]. The short version of the QTF (14 items) was used as a secondary outcome measure to assess the convergent validity of the CAPS-5 in the current study. The internal consistency of the QTF for the recent sample was α = 0.90 and ω = 0.90.

#### Penn state worry questionnaire (PSWQ)

We used the German version [48] of the Penn State Worry Questionnaire [PSWQ; 49]. This self-rating questionnaire was used as a secondary outcome measure to assess the current study’s convergent validity of the CAPS-5. The internal consistency of the PSWQ for the current sample was α = 0.82 and ω = 0.81.

#### Global severity index of the symptom-checklist-90-revised (SCL-90-R)

The German version [50] of the Symptom-Checklist-90-Revised [SCL-90-R; 51] was used here to investigate convergent and discriminant validity. The global severity index (GSI) reflects the overall distress caused by psychological symptoms during the past week, and thus, it was used to assess the convergent validity.

### Questionnaires used for the investigation of the divergent validity of the German CAPS-5

#### Subscales of the symptom-checklist-90-revised (SCL-90-R)

Other subscales of the SCL-90-R ((1) somatization (SOM); (2) obsession-compulsion (OBS); (3) interpersonal sensitivity (INT); (4) phobic anxiety (PHO); (5) paranoid ideation (PAR); and (6) psychoticism (PSY)) were used to determine the divergent validity of the CAPS-5. The internal consistency of the GSI for the current sample was α = 0.98 and ω = 0.98.

Internal consistencies of the relevant SCL-90-R subscales for the current sample were as follows: SOM: α = 0.91 and ω = 0.91; OBS: α = 0.92 and ω = 0.92; INT: α = 0.92 and ω = 0.92; PHO: α = 0.90 and ω = 0.91; PAR: α = 0.85 and ω = 0.86; and PSY: α = 0.88 and ω = 0.88.

#### Balanced inventory of desirable responding (BIDR)

The German version [52] of the balanced inventory of desirable responding (BIDR) is a self-rating questionnaire for assessing socially desirable responses [53, 54]. The BIDR consists of two subscales: (1) self-deceptive enhancement (BIDR-S) and (2) impression management (BIDR-I). It was used to determine the divergent validity of the CAPS-5. The internal consistency of the self-deceptive enhancement subscale was α = 0.74 and ω = 0.73. The internal consistency of the impression management subscale was α = 0.60 and ω = 0.60.

### Statistical analyses

An item analysis was first conducted to investigate the mean, standard deviation, skewness, and kurtosis of the sum score of the German CAPS-5 and the scores of its items. The internal consistency of the CAPS-5 and its subscales was also investigated. Cronbach’s alpha was used to ensure comparability with other studies regarding the CAPS-5. However, considering the potential bias of Cronbach’s α for scales with relatively low numbers of items, the more robust structure equation modeling-based measure of McDonald’s ω [55–57] was also reported. The internal consistency (α/ω) is interpreted as follows: α/ω < 0.50: unacceptable; α/ω < 0.60: poor; α/ω < 0.70: questionable; α/ω < 0.80: acceptable; α/ω < 0.90: good; and α/ω ≥ 0.90: excellent. In the next step, the factorial structure of the German CAPS-5, following the symptom clusters of PTSD in the DSM-5, was investigated by using a confirmatory factorial analysis (CFA). The CFA was conducted using R [version 4.3.1, 58] and the package lavaan [version 0.6–16; 59] for structural equation modeling. The sample size of *N* = 288 has sufficient power given the number of factors in the model (*N* = 4) [60]. As a correction method for the non-normality and heteroscedasticity of the data, the Satorra-Bentler correction was used [61]. The goodness of fit of the German CAPS-5 according to the DSM-5 PTSD model was assessed according to the following fit indices: the relative χ^2^ (χ^2^/*df*), the root-mean-square-error-of-approximation (RMSEA) including the 90% confidence interval (90%-CI), the comparative-fit-index (CFI), the Tucker–Lewis index (TLI), and the standardized root-mean-square residual (SRMR). These fit indices can be interpreted as follows: a relative χ^2^, a value of < 3 indicates a good model fit [62, 63]. Regarding the RMSEA, values of < 0.05 indicate a good model fit, whereas values between < 0.08 and > 0.05 can be seen as a reasonable fit [64]. In the case of the CFI and the TLI, values > 0.90 indicate an adequate fit, whereas values > 0.95 indicate a good fit [65, 66]. For the SRMR, values of < 0.09 indicate a good model fit [67].

The CFA was conducted to test the factorial structure of the German CAPS-5 based on the four-factor model of PTSD as defined in the DSM-5, which includes the clusters: (B) intrusion, (C) avoidance, (D) negative alterations in cognition and mood, and (E) hyperarousal. This structure is also reflected in the original CAPS-5 development and psychometric validation by Weathers et al. [13] and has been used in prior international validations [13, 15, 17, 68].

We chose this model a priori because it is the theoretically grounded and diagnostic-based structure embedded in the CAPS-5 interview format and scoring algorithm. This alignment with the DSM-5 diagnostic criteria ensures both clinical relevance and comparability with other CAPS-5 validation studies.

The test-retest reliability was assessed by conducting intraclass correlation (ICC) analyses, including the 95%-confidence interval, for the German CAPS-5 sum score between the CAPS-5 interviews at T1 and T2 (1 week after T1). For the subscales, Cohen’s Κ was calculated for agreement of diagnoses between the CAPS-5 interviews at T1 and T2. ICC coefficients were interpreted according to Landis & Koch [69] as follows: < 0: poor; < 0.2: slight; < 0.4: fair; < 0.6: moderate; < 0.8: substantial; < 1: almost perfect. The interrater reliability regarding meeting the criteria for PTSD was analyzed by calculating Cohen’s Κ coefficients between the actual assessment of CAPS-5 at T1 and the independent rating of the audio recording of the interview at T1. The ICC, including the 95%-confidence interval, was calculated to investigate the German CAPS-5 sum score agreement between the CAPS-5 interviews at T1 and the independent rating of the audio recording of the interview at T1. The construct validity of the German CAPS-5 was investigated using Spearman’s rank correlation analyses.

Finally, the diagnostic accuracy (true-positive (TP), true-negative (TN), false-positive (FP), and false-negative (FN)) of the German CAPS-5 was calculated using a receiver operating characteristics curve analysis (ROC) with a sum score of the PCL-5 ≥ 33 as the external criterion [24]. These diagnostic accuracy results were used to calculate the sensitivity and specificity. Furthermore, the receiver operating characteristics, AUC, positive likelihood ratio (PLR; sensitivity/(TP + FN)), negative likelihood ratio (NLR; specificity/(TN + FP)), positive predictive value (PPV; TP/(TP + FP)), and negative predictive value (NPV; TN/(TN + FN)) were calculated. The AUC can be interpreted as follows: ≥ 0.60: poor, ≥ 0.70: fair, ≥ 0.80: good, and ≥ 0.90: excellent [70]. Furthermore, a cut-off score for the CAPS-5 was calculated using ROC analyses with a sum score of PCL-5 ≥ 33 and IES-F > 0 as external criteria. The Youden index was used to identify the optimal cut-off values.

The sample size was determined based on both theoretical recommendations and an a priori power analysis [see 23]. Specifically, G*Power (v3.1.9.3) was used to calculate the required sample size for a multivariate analysis of variance (MANOVA) with three independent groups and 23 dependent variables. Assuming a small effect size (*f* = 0.10), an alpha level of 0.05, and a power of 0.80, the analysis indicated a required sample size of *n* = 168. To account for potential dropouts and incomplete data (estimated at 30%), a target of *n* = 219 was set.

Our final sample of *N* = 288 exceeds this threshold and is in line with recommendations for psychometric validation studies. Simulation studies suggest that a sample of 200–300 participants is sufficient for confirmatory factor analysis (CFA) with 4–6 latent variables [60]. Furthermore, this sample size meets published standards for evaluating diagnostic accuracy using ROC analyses [71]. Thus, the study is considered sufficiently powered to support all planned analyses.

## Results

### Descriptive statistics for the German CAPS-5

Table 3 shows that the mean scores per item of the CAPS-5 were relatively high. For example, the lowest mean score with *M* = 0.37 (*SD* = 0.81) was observed for item E2 (“taking more risks or doing things that might have caused harm”), whereas the highest mean score with *M* = 2.07 (*SD* = 1.24) was observed for item B1 (“having unwanted memories of the event while awake”). Between 13.8% (item E2) to 74.8% (item B4) of the participants had severity ratings of 2 or higher (Table 3).

**Table 3:** Descriptive statistics for the German CAPS-5 items

Symptom item	M	SD	Min	Max	skewness	kurtosis	≥ 2 crit. %
B1 (intrusive memories)	2.07	1.24	0	4	-0.46	-0.79	74.0
B2 (distressing dreams)	1.68	1.45	0	4	0.01	-1.51	58.9
B3 (dissociative reactions)	1.02	1.22	0	4	0.69	-0.93	39.0
B4 (psychological distress)	2.06	1.07	0	4	-0.49	-0.42	74.8
B5 (physiological reactions)	1.92	1.25	0	4	-0.37	-1.03	71.1
C1 (avoidance of internal reminders)	1.90	1.35	0	4	-0.29	-1.28	66.3
C2 (avoidance of external reminders)	1.73	1.33	0	4	-0.12	-1.32	61.8
D1 (negative alterations in cognitions)	0.71	1.10	0	4	1.17	-0.20	26.0
D2 (negative beliefs)	1.53	1.35	0	4	0.13	-1.41	55.7
D3 (distorted cognitions)	1.04	1.22	0	4	0.76	-0.73	33.7
D4 (negative emotional state)	1.99	1.19	0	4	-0.47	-0.74	74.4
D5 (dimished interest)	1.96	1.35	0	4	-0.34	-1.25	66.7
D6 (feelings of detachment)	1.42	1.40	0	4	0.33	-1.38	48.4
D7 (inability to experience positive emotions)	1.84	1.36	0	4	-0.16	-1.30	65.5
E1 (irritable behavior)	1.37	1.10	0	4	0.20	-0.83	49.2
E2 (reckless behavior)	0.37	0.81	0	4	2.07	2.95	13.8
E3 (hypervigilance)	1.72	1.32	0	4	-0.10	-1.38	58.9
E4 (startle response)	1.24	1.17	0	4	0.37	-1.02	47.2
E5 (problems with concentration)	1.80	1.25	0	4	-0.31	-1.30	64.6
E6 (sleep disturbance)	2.13	1.36	0	4	-0.49	-1.10	70.3

*N* = 269; *M* = Mean; *SD* = standard deviation; ≥ 2 crit. % = percentage of participants with a severity rating of 2 or higher

Table 4 describes the descriptive statistics of the subscales of the German CAPS-5 and the CAPS-5 sum score for the current sample. Of all participants, 74.8% (subscale C: avoidance) and 84.5% (subscale B: re-experiencing) fulfilled the cluster criteria. Furthermore, 65.9% of the participants fulfilled all the required criteria of PTSD according to CAPS-5.

**Table 4:** Descriptive statistics for the German CAPS-5 subscales and sum scale

Symptom cluster	M	SD	Min	Max	skewness	kurtosis	fulf. crit. %
Cluster B (re-experiencing)	8.72	4.98	0	19	-0.25	-0.89	84.5
Cluster C (avoidance)	3.61	2.40	0	8	-0.15	-1.12	74.8
Cluster D (negative alterations in cognitions and mood)	10.44	6.35	0	24	-0.09	-1.02	80.1
Cluster E (hyperarousal)	8.58	4.71	0	21	-0.08	-0.66	80.1
CAPS-5 sum	31.36	16.31	0	63	-0.30	-0.78	65.9

*N* = 269; *M* = Mean; *SD* = standard deviation; fulf. crit. % = percentage of participants with the fulfilled criterion for cluster or PTSD diagnosis

### Internal consistency

The internal consistency for the full scale of the German CAPS-5 was excellent, with α = 0.93 and ω = 0.93. The internal consistencies were lower for the subscales of the CAPS-5. The analyses showed good internal consistency of cluster B (re-experiencing): α = 0.86 and ω = 0.86. The internal consistency of cluster C (avoidance) was acceptable with α = 0.76. Due to only two items, ω was not possible to calculate for cluster C. Furthermore, the internal consistency of cluster D (cognitions and mood symptoms) was good, with α = 0.82 and ω = 0.84. Finally, the internal consistency of cluster E (hyperarousal) was acceptable with α = 0.73 and ω = 0.75.

### Test-retest reliability

The test-retest reliability was analyzed by comparing the fulfillment of the PTSD diagnosis according to the DSM-5 between T1 and T2. Furthermore, the sum scores of the CAPS-5 subscales and the CAPS-5 sum score were compared between T1 and T2. Therefore, the data of *n* = 87 participants who participated in both measurements were used, and all required data were available.

We next evaluated the PTSD diagnosis between T1 and T2. The analyzes revealed a Κ = 0.70 (T1 + T2: no PTSD: *n* = 21; T1: no PTSD, T2: PTSD: *n* = 7; T1 PTSD, T2: no PTSD: *n* = 4; T1, T2: PTSD: *n* = 55).

Analyses of the agreement between both measurements for the German CAPS-5 sum score revealed a nearly perfect agreement with an ICC = 0.92 (0.87–0.95). The agreements for all subscale scores of the German CAPS-5 showed nearly perfect agreement (cluster B: ICC = 0.89 (0.83–0.93); cluster C: ICC = 0.87 (0.80–0.92); cluster D: ICC = 0.85 (0.78–0.90); cluster E: ICC = 0.90 (0.84–0.93)).

### Interrater reliability

The interrater reliability was based on data of *n* = 113 independent ratings of the audio record of the interview at T1. The interrater reliability regarding the existence of the PTSD diagnosis was analyzed by calculating Cohen’s Κ coefficients between the actual assessment of CAPS-5 at T1 and the independent rating of the audio record of the interview at T1. Cohen’s Κ for the PTSD diagnoses between the interview at T1 and the independent rating of the audio record of the interview at T1 was Κ = 1.0. The interrater reliability for the German CAPS-5 sum score and the subscales was almost perfect: (CAPS-5 sum score: ICC = 0.96 (0.93–0.97); cluster B: ICC = 0.98 (0.96–0.99); cluster C: ICC = 0.92 (0.87–0.95); cluster D: ICC = 0.96 (0.94–0.98), and cluster E: ICC = 0.96 (0.93–0.97)).

### Confirmatory factorial analysis

**Fig. 1:**
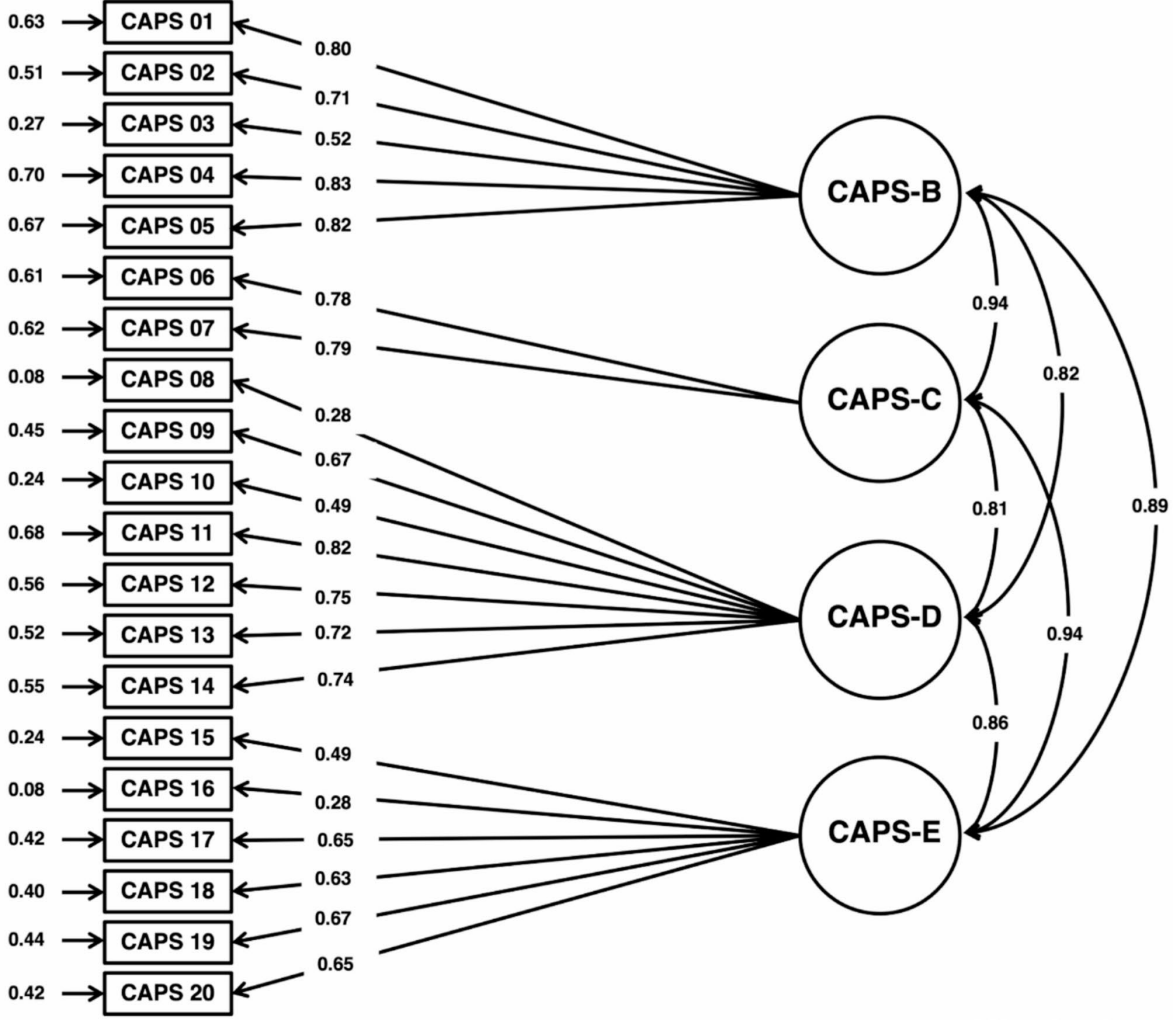
CFA results for data collected using the German CAPS-5 to test the DSM-5 model of PTSD

Overall, the analysis of the factorial structure of the German CAPS-5 showed an adequate to good model fit. The relative χ^2^ = 2.14 (χ^2^: 351.16 / *df*: 164), the CFI (= 0.93), the TLI (= 0.92), and the SRMR (= 0.05) indicate a good model fit, whereas the RMSEA = 0.06 (95%-CI = 0.05–0.07) can be seen as an indicator of an adequate model fit. Further details on the CFA results can be found in Fig. 1.

### Construct validity

#### Convergent validity

The German CAPS-5 sum and subscales scores were significantly associated with most other scales (Table 5). The highest significant associations were found between the German CAPS-5 and its subscales and other measures of PTSD symptoms (PCL-5, SkPTBS, IES-R-F, and IES-R-D). As expected, the associations between the German CAPS-5 sum score and the subscales and borderline associated thoughts and feelings (QTF) were significant but slightly lower. Only the association between the QTF and the subscale of cluster D (cognitions and mood symptoms) of the German CAPS-5 was insignificant. Significant positive associations were observed between the German CAPS-5 sum score and worries (measured with the PSWQ). Also, the CAPS-5 subscales B (re-experiencing), C (avoidance), and D (cognitions and mood symptoms) showed significant positive associations with the PSWQ.

**Table 5:** Spearman rank correlation analyses between the German CAPS-5 and its subscales and questionnaires to measure construct validity

		PCL-B			PCL-C		PCL-D		PCL-E		PCL sum		SkPTBS		IES-F		IES-D		QTF		PSWQ		DES-20	
Cluster B (re-experiencing)		0.746*** (*n* = 249)			0.613*** (*n* = 248)		0.564*** (*n* = 248)		0.645*** (*n* = 247)		0.709*** (*n* = 250)		0.417*** (*n* = 100)		0.607*** (*n* = 144)		0.592*** (*n* = 144)		0.357*** (*n* = 148)		0.255** (*n* = 150)		0.569*** (*n* = 245)	
Cluster C (avoidance)		0.577*** (*n* = 249)			0.675*** (*n* = 248)		0.541*** (*n* = 248)		0.631*** (*n* = 247)		0.656*** (*n* = 250)		0.453*** (*n* = 100)		0.506*** (*n* = 144)		0.521*** (*n* = 144)		0.281*** (*n* = 148)		0.237** (*n* = 150)		0.447*** (*n* = 245)	
Cluster D (negative alterations in cognitions and mood)		0.553*** (*n* = 249)			0.547*** (*n* = 248)		0.759*** (*n* = 248)		0.670*** (*n* = 247)		0.713*** (*n* = 250)		0.787*** (*n* = 100)		0.464*** (*n* = 144)		0.494*** (*n* = 144)		0.558*** (*n* = 148)		0.393*** (*n* = 150)		0.618*** (*n* = 245)	
Cluster E (hyperarousal)		0.525*** (*n* = 249)			0.522*** (*n* = 248)		0.673*** (*n* = 248)		0.679*** (*n* = 247)		0.610*** (*n* = 250)		0.406*** (*n* = 100)		0.426*** (*n* = 144)		0.438*** (*n* = 144)		0.234** (*n* = 148)		0.197 (*n* = 150)		0.446*** (*n* = 245)	
CAPS sum		0.664*** (*n* = 249)			0.638*** (*n* = 248)		0.705*** (*n* = 248)		0.738*** (*n* = 247)		0.758*** (*n* = 250)		0.641*** (*n* = 100)		0.605*** (*n* = 144)		0.614*** (*n* = 144)		0.462*** (*n* = 148)		0.350*** (*n* = 150)		0.600*** (*n* = 245)	

	STAI-S		STAI-T	SCL-S		SCL-O		SCL-I		SCL-Ph		SCL-Pa		SCL-Ps		SCL-GSI		BDI-II		BIDR-S		BIDR-I	
CAPS-B	0.390*** (*n* = 150)		0.323*** (*n* = 150)	0.538*** (*n* = 250)		0.550*** (*n* = 250)		0.512*** (*n* = 250)		0.663*** (*n* = 250)		0.423*** (*n* = 250)		0.529*** (*n* = 250)		0.642*** (*n* = 248)		0.424*** (*n* = 145)		-0.220** (*n* = 149)		-0.051 (*n* = 149)	
CAPS-C	0.390*** (*n* = 150)		0.339*** (*n* = 150)	0.441*** (*n* = 250)		0.504*** (*n* = 250)		0.495*** (*n* = 250)		0.613*** (*n* = 250)		0.372*** (*n* = 250)		0.454*** (*n* = 250)		0.569*** (*n* = 248)		0.365*** (*n* = 145)		-0.209** (*n* = 149)		-0.080 (*n* = 149)	
CAPS-D	0.530*** (*n* = 150)		0.515*** (*n* = 150)	0.522*** (*n* = 250)		0.647*** (*n* = 250)		0.693*** (*n* = 250)		0.638*** (*n* = 250)		0.640*** (*n* = 250)		0.669*** (*n* = 250)		0.737*** (*n* = 248)		0.609*** (*n* = 145)		-0.423** (*n* = 149)		-0.016 (*n* = 149)	
CAPS-E	0.394*** (*n* = 150)		0.281*** (*n* = 150)	0.452*** (*n* = 250)		0.524*** (*n* = 250)		0.443*** (*n* = 250)		0.627*** (*n* = 250)		0.393*** (*n* = 250)		0.398*** (*n* = 250)		0.553*** (*n* = 248)		0.327*** (*n* = 145)		-0.134* (*n* = 149)		-0.036 (*n* = 149)	
CAPS sum	0.515*** (*n* = 150)		0.446*** (*n* = 150)	0.547*** (*n* = 250)		0.637*** (*n* = 250)		0.618*** (*n* = 250)		0.721*** (*n* = 250)		0.533*** (*n* = 250)		0.591*** (*n* = 250)		0.714*** (*n* = 248)		0.550*** (*n* = 145)		-0.307** (*n* = 149)		-0.040 (*n* = 149)	

*CAPS-B* CAPS-5 re-experiencing subscale, *CAPS-C* CAPS-5 avoidance subscale, *CAPS-D* CAPS-5 cognitions and mood symptoms subscale, *CAPS-E* CAPS-5 hyperarousal subscale, *CAPS sum* CAPS-5 sum score, *PCL-B* PCL-5 re-experiencing subscale, *PCL-C* PCL-5 avoidance subscale, *PCL-D* PCL-5 cognitions and mood symptoms subscale, *PCL-E* PCL-5 hyperarousal subscale, *PCL sum* PCL-5 sum score, *IES-F* Impact of Event Scale-Revised frequency version, *IES-D* Impact of Event Scale-Revised distress version, *QTF* Questionnaire of Thoughts and Feelings, *PSWQ* Penn State Worry Questionnaire, *DES-20* Dissociative Experiences Scale – 20 items version, *STAI-S* State-Trait Anxiety Inventory – State subscale, *STAI-T* State-Trait Anxiety Inventory –Trait subscale, *SCL-S* Symptom-Checklist-90-Revised Somatization subscale, *SCL-O* Symptom-Checklist-90-Revised Obsessive-Compulsive subscale, *SCL-I* Symptom-Checklist-90-Revised Interpersonal Sensitivity subscale, *SCL-Ph* Symptom-Checklist-90-Revised Phobic Anxiety subscale, *SCL-Pa* Symptom-Checklist-90-Revised Paranoid Ideation subscale, *SCL-Ps* Symptom-Checklist-90-Revised Interpersonal Psychoticism, *SCL-GSI* Symptom-Checklist-90-Revised Global Severity, *BDI-II* Beck Depression Inventory-Revised, *BIDR-S* Balanced Inventory of Desirable Responding self-deceptive enhancement subscale, *BIDR-I* Balanced Inventory of Desirable Responding impression management subscale

In contrast, subscale E (hyperarousal) was not significantly associated with the PSWQ. As expected, the CAPS-5 sum score and the scores of all subscales showed significant positive associations with dissociation symptoms (DES-20), depression (BDI-II), state (STAI-S), and trait (STAI-T) anxiety. As also expected, the German CAPS-5 sum score and its subscales showed significantly positive associations with the SCL-90-R-GSI.

#### Divergent validity

Contrary to our expectations, all other subscales of the SCL-90-R showed significantly positive associations with the German CAPS-5 sum score and its subscales (Table 5). The social desirability scale’s impression management subscale (BIDR-I) was not significantly associated with the German CAPS-5 sum score and its subscales. However, contrary to our expectations, the self-deceptive enhancement (BIDR-S) subscale showed significant negative associations with the German CAPS-5 sum score and its subscales.

#### Diagnostic accuracy

**Fig. 2:**
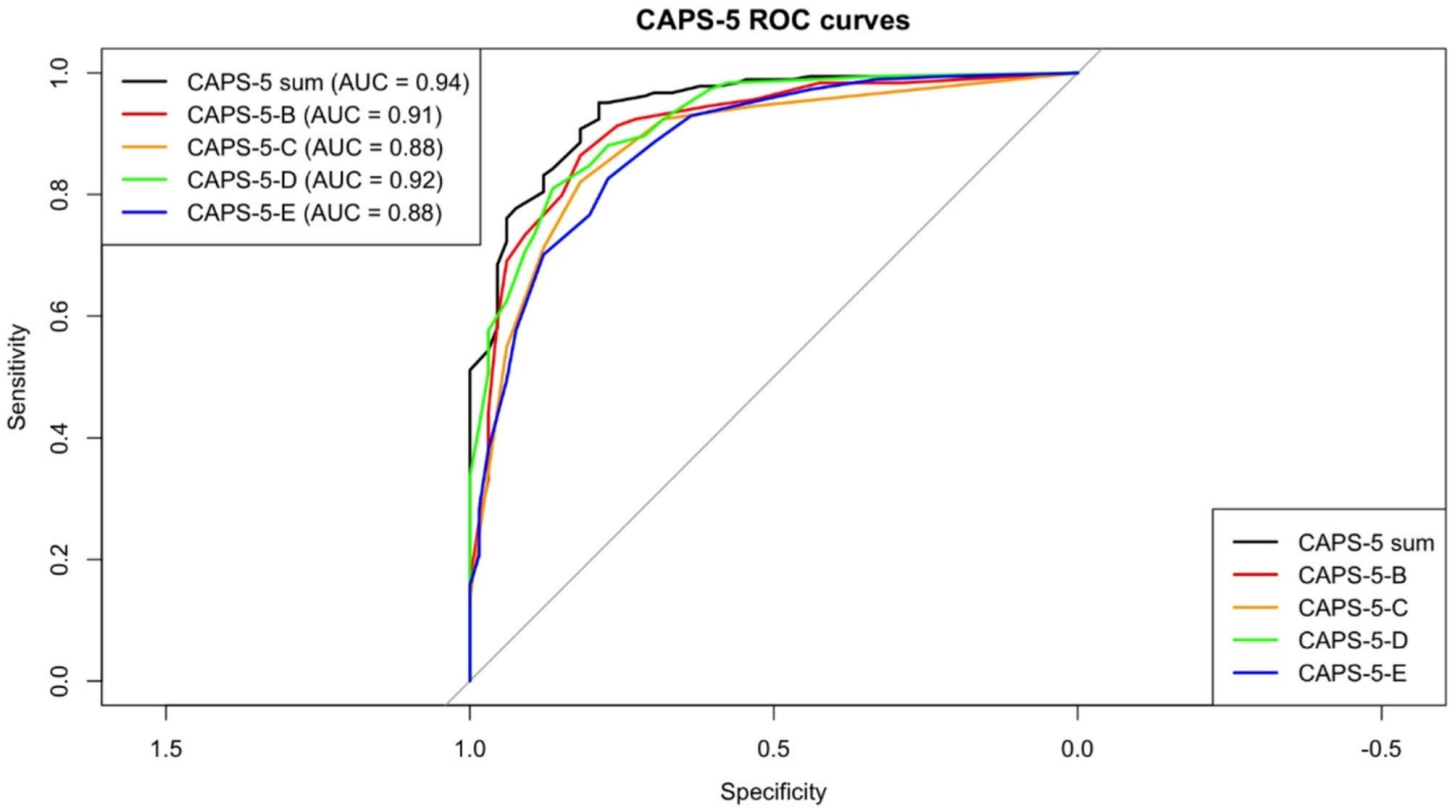
Receiver operating characteristic (ROC) curves for the German CAPS-5 total score and subscales (criterion: PCL-5 ≥ 33)

ROC analysis with a sum score of PCL-5 ≥ 33 as the external criterion was used to investigate the diagnostic accuracy of the German CAPS-5 sum score and the CAPS-5 subscales (see Table 6). The ROC analyses showed excellent diagnostic accuracy for the German CAPS-5 (AUC = 0.94) and the subscales for cluster B (re-experiencing; AUC = 0.91) and cluster D (cognitions and mood symptoms; AUC = 0.92). The ROC analyses showed good diagnostic accuracy for the subscales of cluster C (avoidance; AUC = 0.88) and cluster E (hyperarousal; AUC = 0.88). Thus, the ROC analyses confirmed excellent diagnostic performance of the German CAPS-5 total score and good to excellent accuracy for all subscales (see Fig. 2).

**Table 6:** Diagnostic parameters of the CAPS-5 total scale and subscales (ROC analysis, criterion: PCL-5 ≥ 33)

Symptom cluster	AUC	Cut-off	sen	spe	PPV	NPV	PLR	NLR	Youden Index
Cluster B (re-experiencing)	0.91	≥ 7	0.86	0.82	0.93	0.68	4.75	0.17	0.68
Cluster C (avoidance)	0.88	≥ 3	0.82	0.82	0.93	0.62	4.51	0.22	0.64
Cluster D (negative alterations in cognitions and mood)	0.92	≥ 9	0.81	0.86	0.94	0.62	5.94	0.22	0.67
Cluster E (hyperarousal)	0.88	≥ 7	0.83	0.77	0.91	0.61	3.64	0.23	0.60
CAPS-5 sum	0.94	≥ 39	0.83	0.82	0.93	0.63	4.61	0.21	0.61

*AUC* area under the curve, *sen* sensitivity, *spe* specificity, *PPV* positive predictive value, *NPV* negative predictive value, *PLR* positive likelihood ratio, *NLR* negative likelihood ratio

**Fig. 3:**
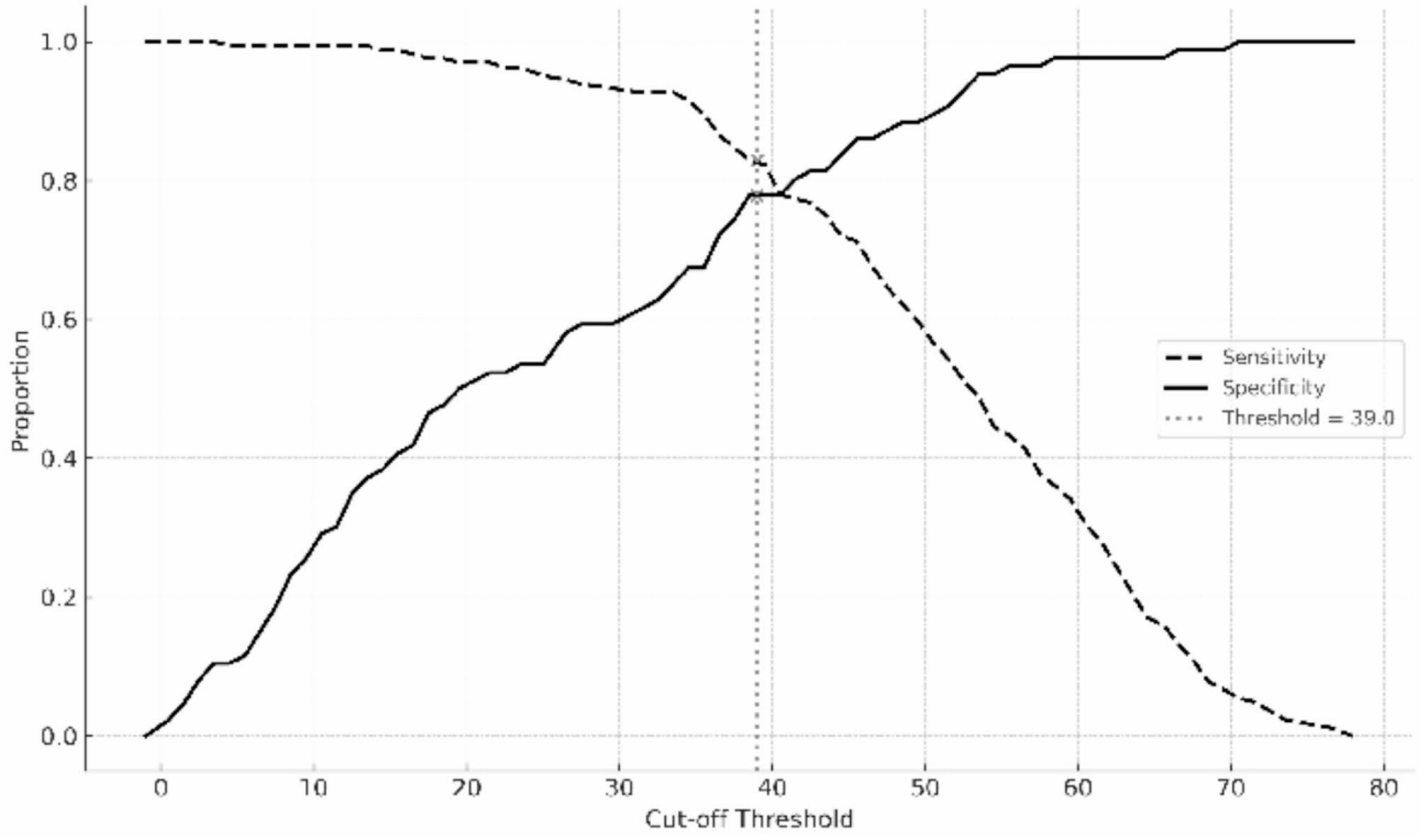
Sensitivity and specificity across cut-off thresholds for the German CAPS-5 total score (criterion: PCL-5 ≥ 33)

The ROC data led to calculations of sensitivity, specificity, PLR, NLR, PPV, and NPV of the German CAPS-5 sum score. The results showed a sensitivity of 0.83 and a specificity of 0.82. Furthermore, these analyses revealed a PPV = 0.93 and an NPV = 0.63, which resulted in a PLR = 4.61 and an NLR = 0.21. Finally, the optimal cut-off for the CAPS-5 total score was determined by examining the trade-off between sensitivity and specificity across the full range of possible thresholds (see Fig. 3). Based on the highest Youden Index, a cut-off of ≥ 39 yielded a sensitivity of 0.83 and a specificity of 0.82 when using the PCL-5 cut-off of ≥ 33 as the external criterion. The results of the ROC analysis for the subscales of the CAPS-5 can be found in Table 6. The ROC analysis with a score of the IES-F > 0 as an external criterion revealed a cut-off score of ≥ 42 (sensitivity = 0.65; 1-specificity = 0.20; Youden Index = 0.45) for the German CAPS-5.

## Discussion

We presented the results of the psychometric properties of the German CAPS-5 in routine clinical settings. The descriptive statistics for the items of the German CAPS-5, the subscales of the German CAPS-5, and the overall CAPS-5 were investigated. In addition, the internal consistencies, test-retest reliability, interrater reliability, construct validity, and diagnostic accuracy of the German CAPS-5 and its subscales were also investigated. The German CAPS-5 was a structured diagnostic interview with good to excellent internal consistency, test-retest reliability, interrater reliability, construct validity, and diagnostic accuracy. Most of our hypotheses [see 23] were supported, and the results of this study support the value of the CAPS-5 for diagnosing PTSD in clinical and scientific settings.

The descriptive statistics of the items of the CAPS-5 showed relatively high mean scores. The participants showed a mean score of > 1 for most items. More than half of the participants fulfilled the criterion of a score ≥ 2 for 14 of 20 items of the CAPS-5. The lowest means were observed for items B3 (“dissociative reactions”), D1 (“inability to remember an important aspect”), and E2 (“reckless or self-destructive behavior”). These results align with Weathers et al. [13], who also reported the lowest means for these items. In other studies, these items also showed relatively low mean scores and were excluded from confirmatory factor analyses [13, 15, 18, 72]. The mean sum scores of the German CAPS-5 and its subscales were similar to the results of other studies [13, 15, 72]. Most participants (≥ 74.8%) fulfilled the criteria of at least one subscale of the CAPS-5 according to the DSM-5 (Table 3). However, in the current sample, the diagnostic criteria of PTSD according to the DSM-5 were only fulfilled by 65.9% of the participants. These results showed that the current cohort had an overall high symptom severity and was highly mentally stressed, which are basic assumptions for investigating a diagnostic tool. However, these results represent an apparent variability and a relatively high percentage of TN cases, facilitating a differentiated analysis of the diagnostic accuracy of the German CAPS-5.

The internal consistencies of the German CAPS-5 were excellent and acceptable to good for the subscales of the German CAPS-5. Thus, the picture regarding the internal consistencies of the German CAPS-5 is consistent with studies regarding the original version [13] and other translations of the CAPS-5 [15, 16].

The other study on the German CAPS-5 showed comparable internal consistencies [19]. However, a closer look at the internal consistencies showed good coefficients for the subscales of cluster B (re-experiencing) and cluster D (cognitions and mood symptoms). In contrast, the internal consistencies for the subscales of cluster C (avoidance) and cluster E (hyperarousal) were acceptable. These differences in the internal consistencies for the subscales were also reported in other studies of the CAPS-5 [13, 15, 16, 19]. The cluster C subscale’s relatively lower and acceptable internal consistency is due to the number of items in this subscale. Scales with only two items tend to show a lower internal consistency [73]. The heterogeneity of the items of the cluster E subscale and the potential loading of these items on different factors [13, 72] could explain the relatively lower internal but acceptable consistency of the cluster E subscale.

The results of the CFA overall showed an adequate to good model fit of the German CAPS-5 factorial structure according to the DSM-5 criteria of PTSD. Thus, the instrument can be used in clinical routines and research to diagnose PTSD in line with the PTSD criteria according to the DSM-5. These results can also be seen as an indicator of sufficient construct validity [74]. Although the CFA supported an adequate to good fit for the assumed four-factor DSM-5 model, the high inter-factor correlations (*r* > 0.90) suggest substantial conceptual overlap between the clusters. This finding indicates that the four-factor structure should be interpreted as provisional. Future research should test and compare alternative models, such as bifactor models, reduced-dimensional (e.g., 2- or 3-factor), or unidimensional structures, to better determine the latent structure of PTSD symptoms in German-speaking populations [15, 24, 75–80]. These models may offer a more parsimonious and psychometrically robust representation of PTSD symptomatology.

The test-retest and interrater reliability of the German CAPS-5 and its subscales revealed excellent reliability coefficients. These coefficients were comparable to the results regarding the test-retest reliability and interrater reliability of the CAPS-5 [13, 15, 16] and slightly higher than the German CAPS-5 [19]. However, the number of participants who participated at both measurement points was relatively low (30.2%). Only 39.2% of all interviews were independently rated for investigating interrater reliability. Thus, further studies regarding the test-retest and interrater reliability of the German CAPS-5 are needed to provide more robust results.

Regarding the construct validity of the German CAPS-5, the results confirmed the hypotheses regarding the convergent validity of the German CAPS-5 and its subscales. The German CAPS-5 and its subscales showed strong correlations with other measures of PTSD (PCL-5, IES-R) and cPTSD (SkPTBS), as well as measures of psychopathological constructs that are highly associated with PTSD (e.g., anxiety or depression). Measures of more divergent psychopathological constructs (e.g., worries or borderline personality disorder-associated thoughts and feelings) were also significant but less strongly associated with the CAPS-5 scores. Indeed, the hypotheses regarding the divergent validity of the German CAPS-5 and its subscales were only partially supported by the results. As expected, parts of the socially desirable responses (impression management) were not significantly associated with the German CAPS-5 sum score and its subscales. The self-deceptive enhancement aspect of the socially desirable responses revealed significant and negative associations with the German CAPS-5 sum score and its subscales. However, contrary to our hypotheses, all subscales of the SCL-90-R showed significant associations with the German CAPS-5 sum score and its subscales. These results underscore the high psychopathological distress of the current sample. In retrospect, the choice of instruments for investigating the divergent validity could have been better. For instance, other study results showed significant positive associations between PTSD and somatization [e.g., 13, 81]. Future studies should focus more on measures that are less associated with psychopathology (e.g., personality measures).

Following the MTMM framework, we categorized the observed correlation coefficients to evaluate construct validity. High monotrait-heteromethod correlations between CAPS-5 subscales and their corresponding PCL-5 scales (e.g., CAPS-B and PCL-B: *r* = 0.746) demonstrate strong convergent validity, suggesting that both instruments validly capture the same PTSD symptom clusters despite differing methods (interview vs. questionnaire).

In contrast, correlations between CAPS-5 scales and unrelated constructs (e.g., CAPS-B and BIDR-I: *r* = –0.051; CAPS-D and PSWQ: *r* = 0.393) were substantially lower, providing evidence for discriminant validity.

Monotrait–monomethod coefficients were not represented in this table but are reflected in the high internal consistency (Cronbach’s α) and test–retest reliabilities (ICCs) reported elsewhere in the manuscript. Furthermore, low heterotrait–heteromethod correlations (e.g., CAPS-E and BIDR-I: *r* = –0.036) further support discriminant validity, suggesting that method variance did not dominate the observed relationships.

The ROC analyses showed that the German CAPS-5 is a diagnostic instrument with good to excellent accuracy. Furthermore, these results showed that the German CAPS-5 has high sensitivity and specificity and ensures that persons with PTSD are diagnosed correctly; people without PTSD can also be adequately identified. Thus, the German CAPS-5 is an adequate diagnostic interview for the scientific, clinical, and expert assessment context.

Finally, the results investigating a cut-off score of the German CAPS-5 sum score showed an optimal score of ≥ 40 with both the PCL-5 and the IES-F as external criteria. To our knowledge, this is the first time a cut-off score was calculated for the CAPS-5. It can guide using the CAPS-5 in the scientific, clinical, and expert assessment context. However, this cut-off score should be used cautiously until it is replicated in further studies. Importantly, only self-rating questionnaires were used as external criteria, which could bias the resulting cut-off score. The mean PCL-5 score in this study was higher than the mean score of the CAPS-5 (PCL-5: *M* = 42.2 vs. CAPS-5: *M* = 31.36). Higher mean scores of the PCL-5 versus the CAPS-5 were also formerly reported [24]. This leads to the assumption that self-rating questionnaires of PTSD result in higher scores than structured diagnostic interviews. Thus, further investigations of the CAPS-5 cut-off score with another structured diagnostic interview as an external criterion could help answer this question. However, more detailed studies comparing the CAPS-5 and the PCL-5 are needed.

## Challenges and limitations

This study does have some limitations. First, the current sample showed relatively high symptom severities in the CAPS-5 and its subscales. Accordingly, the results regarding diagnostic accuracy could be biased due to high scores. Furthermore, the sample consists of a relatively high percentage of male participants with a military background. Thus, the results should be generalized with caution. Also, the analyses regarding the divergent validity are limited. Therefore, this part of the construct validity still needs to be answered. Also, we did not include data about comorbid diagnoses or control for other diagnoses that could interfere with PTSD symptoms (e.g., borderline personality disorder). Comorbidity should be considered as a potential covariate that could be relevant for the investigated associations. This should be noted in future studies. Finally, although a wide range of instruments was used to evaluate convergent and discriminant validity according to the MTMM framework, the total number of assessments may have contributed to respondent fatigue. This could have attenuated discriminant validity or inflated shared method variance, especially in self-report-based instruments.

## Conclusion

Overall, the results of this study show good to excellent psychometric properties of the German CAPS-5. The German CAPS-5 has good diagnostic accuracy and differentiates between people with and without PTSD. Thus, the results regarding the psychometric properties of the German CAPS-5 confirm the psychometric properties of the original version of the CAPS-5 and other translations.

## Data Availability

The civilian datasets used and analyzed during the current study will be freely available at https://doi.org/10.23668/psycharchives.12858 from May 2023. The military datasets cannot be shared publicly due to military restrictions. However, these data can be viewed in individual cases after application; please contact the corresponding author for details.
